# Supplementary material for: Extensive Genome Exploration of Clostridium botulinum Group III Field Strains
Source: Microorganisms. 2021 Nov 13;9(11):2347. doi: 10.3390/microorganisms9112347 (PMC8624178; doi:10.3390/microorganisms9112347)
Supplement: Supplementary file 1 [file microorganisms-09-02347-s001.zip › microorganisms-1425374-supplementary.pdf]

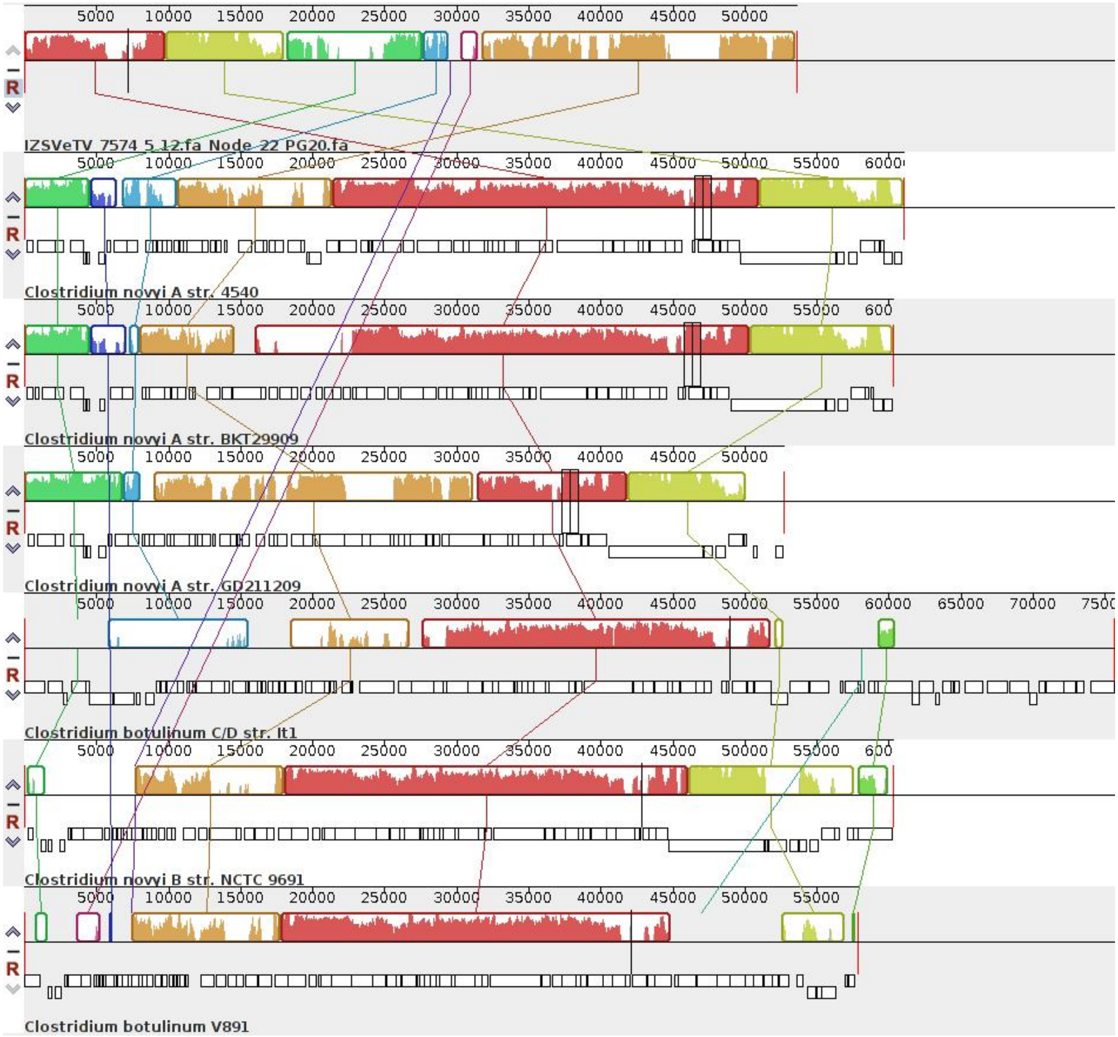

Figure S1. Graphical representation of the alignment between sequences of plasmid categories PG10 and PG20.

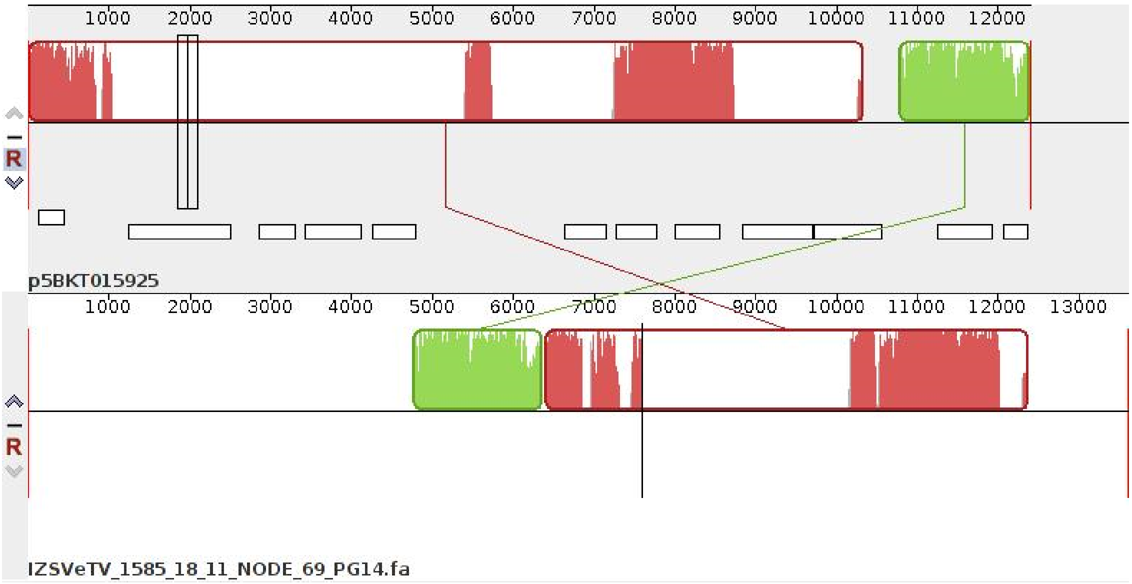

Figure S2. Graphical representation of the alignment between sequences of plas-mid categories PG7 and PG14.
